# Chronic kidney disease correlates with MRI findings of cerebral small vessel disease

**DOI:** 10.1080/0886022X.2021.1873804

**Published:** 2021-01-21

**Authors:** Tingting Yao, Guoping Song, Yuehua Li, Dan Wang

**Affiliations:** Institute of Diagnostic and Interventional Radiology, The Sixth Affiliated People’s Hospital, Shanghai Jiao Tong University, Shanghai, China

**Keywords:** Cerebral small vessel disease, chronic kidney disease, glomerular filtration rate, MRI, Virchow-Robin spaces

## Abstract

**Objective:** Cerebral small vessel disease (CSVD) and chronic kidney disease (CKD) may be part of a multisystem small-vessel disorder. Since the kidney and brain share unique susceptibilities to vascular injury, kidney impairment may be predictive of the presence and severity of CSVD. This study explored the relationship between CSVD and CKD.

**Methods:** Between December 2015 and December 2017 (follow-up 10–20 months) 52 patients with chronic nephritis and CKD were classified into a progressive group (*n* = 17) and stable group (*n* = 35). Age, gender, hypertension, diabetes and smoking were matched between groups. CSVD features of both groups, including enlarged Virchow-Robin spaces (VRS), white matter lesions (WML), lacunar infarcts (LI), and cerebral microbleeds (CMB) were evaluated by magnetic resonance (MR) imaging.

**Results:** WML and CMB in the progressive group were exacerbated at follow-up compared to initial exam (*p* = 0.004 and 0.041, respectively). There was no significant change in VRS, WML, LI, or CMB in the stable group at follow-up compared to initial exam. CMB were significantly different between the progressive group and stable group at follow-up.etimtaed Glomerular filtration rate (eGFR) was significantly correlated with VRS, WML, and CMB at follow-up (*p* = 0.037, 0.041, and 0.009, respectively).

**Conclusions:** Patients with progressive CKD have a higher prevalence and severity of CSVD, which correlates with deterioration of renal function as assessed by decreased eGFR. Thus EGFR may also be of value in the prediction of cerebral small vessel disease.

## Introduction

Chronic kidney disease is often accompanied by metabolic disturbances, disorders of water, electrolytes, and acid-base balance, and pathology of the nervous system. Estimated glomerular filtration rate (eGFR) is used to approximate the degree of renal dysfunction in chronic kidney disease, specifically as an estimate of the extent of functional nephron loss, and as such may be useful to guide the diagnosis and treatment of kidney disease.

The earliest CKD-associated lesion occurring in the nervous system is damage to small blood vessels, including small arteries, arterioles, capillaries, and small veins [1]. These vessels have poor collateral anastomoses, causing increased susceptibility to ischemic or hemorrhagic stroke in the brainstem and deep white matter of the central nervous system. Parenchymal brain lesions are considered to be caused by pathologic changes in small blood vessels, which are collectively a sign of cerebral small vessel disease (CSVD) [2–5]. Features of silent cerebral small vessel disease include enlarged Virchow-Robin spaces (VRS), white matter lesions (WML), lacunar infarcts (LI), and cerebral microbleeds (CMB).

The correlation between CKD and CSVD is mainly due to similar hemodynamics, anatomical, and functional features of the kidney and brain. Unlike most other organs, both the kidneys and the brain are low-impedance terminal organs, which are exposed to high-flow blood shocks throughout the whole cardiac cycle [6]. Similar hemodynamics can be observed in the kidney and brain vessel beds [7,8]. Therefore, CKD-associated pathological changes in small vessels of the kidneys may also exist in small brain arteries [9].

Arteries are exposed to a high pressure, and must maintain strong vascular tone in order to provide a large pressure gradient over a short distance. Ito et al. proposed a very interesting ‘strain of blood vessel hypothesis,’ which they believe can be used as a brain-kidney association mechanism, based on the similar anatomical and functional features of small arteries in these organs [10].

This study aims to explore the progression of small vessel disease in CKD and its relationship to CSVD. Magnetic Resonance (MR) imaging is used to evaluate CSVD progression, as measured by VRS size, white matter lesions (WML), presence of lacunar infarcts (LI), and cerebral microbleeds (CMB).

## Material and methods

### Patients

**Figure 1.**
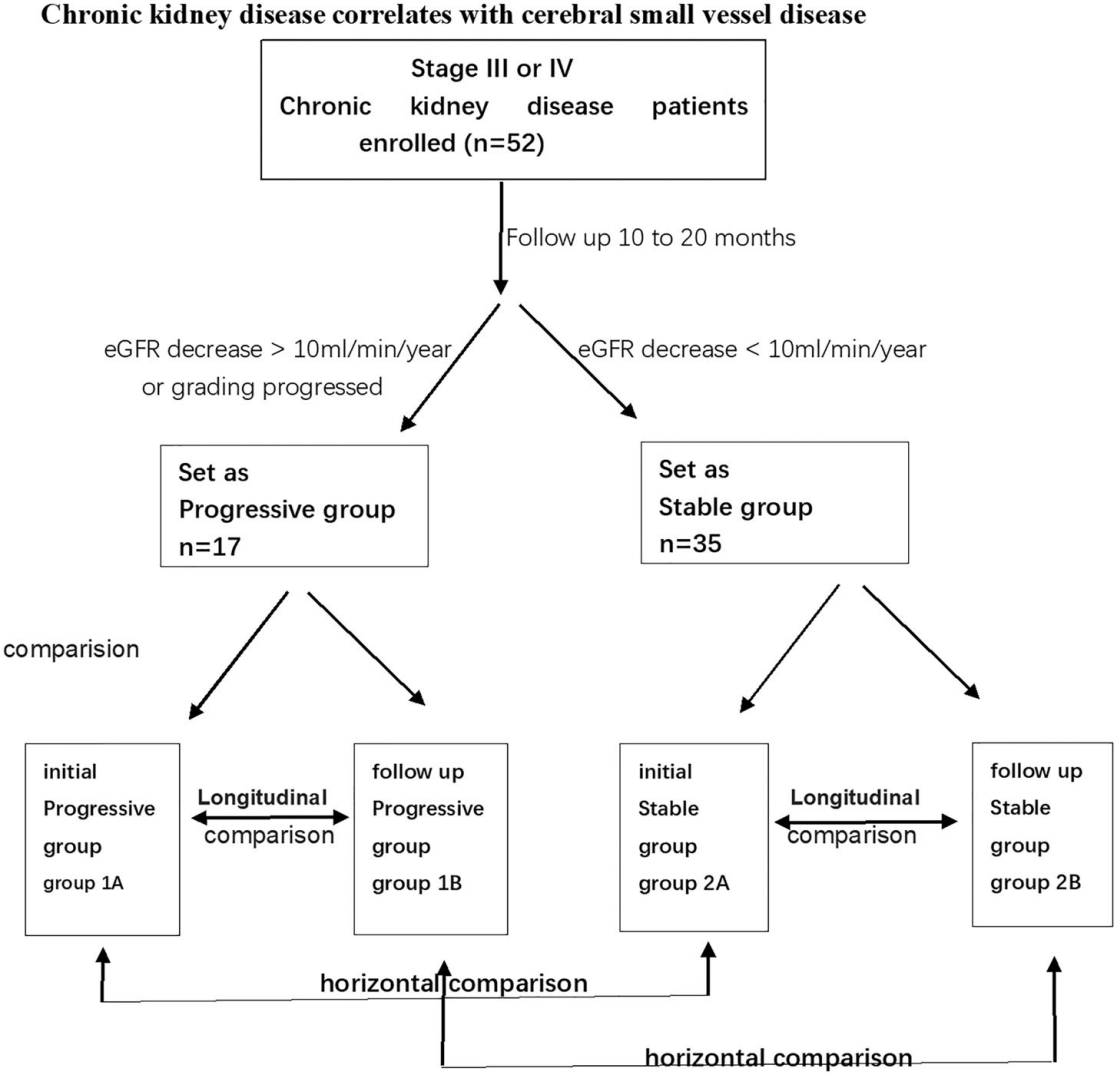
Flow chart of the study design. Fifty two participants with CKD were separated into a progressive group and a stable group. CSVD (cerebral small vessel disease; enlarged VRS/WML/LI/CMB) comparisons were made between the two groups (progressive group at initial exam (group 1A), progressive group at follow-up (group 1B), stable group at initial exam (group 2A), stable group at follow-up (group 2B).

Between December 2015 and December 2017, 55 patients with CKD were enrolled in this study; three were lost to follow-up. Inclusion criteria were as follows: (1) met clinical diagnostic criteria for CKD, stage III or IV, as defined according to the Kidney Disease Outcomes Quality Initiative (KDOQI). CKD is defined as a variety of causes of chronic kidney structure and dysfunction (history of renal damage over 3 months), with or without changes in eGFR, abnormal blood or urine components, and abnormal imaging studies or unexplained decrease in eGFR (<60 mL/min/1.73 m^2^) for more than 3 months; (2) Patients were followed for 10–20 months. eGFR decrease >10 mL/min/year If eGFR decreased by greater than 10 mL/min or CKD severity progressed in stage, patients were assigned to the progressive group [11,12]. If eGFR decreased by less than 10 mL/min, patients were assigned to the stable group (Figure 1, Table 1).

**Table 1.** Clinical data for participants (progressive group and stable group).

	Progressive group	Stable group	Comparisons between two groups *p* value
Number (*N*)	17	35	
Age (years)	52.35 ± 6.99	54.46 ± 8.73	
Gender（male/female）	11/6	19/16	0.476
Proportion who are smokers (%)	7 (41.2%)	12 (28.57%)	0.629
Hypertension (%)	4 (23.53%)	8 (22.86%)	0.956
Diabetes (type 2)	3 (17.65%)	9 (25.71%)	0.517
Total cholesterol (TC) (mmol/L)	5.10±1.01	5.30±0.95	
TG (Triacylglycerol)	1.44±0.46	1.49±0.48	
FBG (fasting blood glucose)	5.63±0.44	5.42±0.48	
Urine erythrocytes	2.529 ± 1.463	1.686 ± 1.491	
Urine protein			
<0.2 g/L	*n* = 0	*n* = 2	
0.2g∼1.0 g/L	*n* = 3	*n* = 7	
1.0–2.0 g/L	*n* = 4	*n* = 8	
2.0–4.0 g/L	*n* = 3	*n* = 12	
>4.0 g/L	*n* = 7	*n* = 6	
eGFR			
Initial eGFR	35.975±11.645	36.765±12.040	0.82338
Follow-up eGFR	24.326±9.217	36.723±11.416	<0.001*
Initial CKD stage			
Stage III	10	21	
Stage IV	7	14	
CKD stage at follow-up			
Stage III	4	25	
Stage IV	13	10	

*Note*: *p < 0.05 was statistically significant.

Written informed consent for the use of data was obtained from patients and volunteers prior to MRI scanning.

### MR examination

All participants underwent MR examinations between December 2015 and December 2017. A 3T MR scanner (MAGNETOM Verio, Siemens Healthcare, Erlangen, Germany) with a 32-channel head coil was used. The imaging sequences included conventional MR sequences (T1- and T2-weighted imaging) as well as DWI (diffusion weighted imaging) and SWI (susceptibility weighted imaging). Protocols were as follows: (1) T2-FOV 250 mm, TR/TE 6000/95 ms, flip angle 150°, matrix 384 × 384, slice thickness 6 mm, distance factor 30%; (2) T1 FLAIR-FOV 250 mm, TR/TE 2,000/9 ms, flip angle 150°, matrix 320 × 320, slice thickness 6 mm, distance factor 30%; and (3) SWI-FOV 230 mm, TR/TE 28/20 ms, flip angle 15°, matrix 320 × 320, resolution 0.76 × 0.76 × 1.2, slice thickness 1.2 mm, distance factor 30%. A group of magnitude, phase, minimum intensity projection (MIP), and SWI images were automatically reconstructed online.

### Image analysis: CSVD evaluation

Silent markers of CSVD include enlarged VRS, white matter lesions (WMLs), lacunar infarcts (LIs), and cerebral microbleeds (CMBs). Two neuroradiologists (with six and nine years of experience, respectively) were highly trained before they reviewed images, and blinded to all clinical details, and recorded the locations and VRS, WML, LI, and CMB ratings. Reliability was assessed on both intra- and interobserver level.

**Figure 2.**
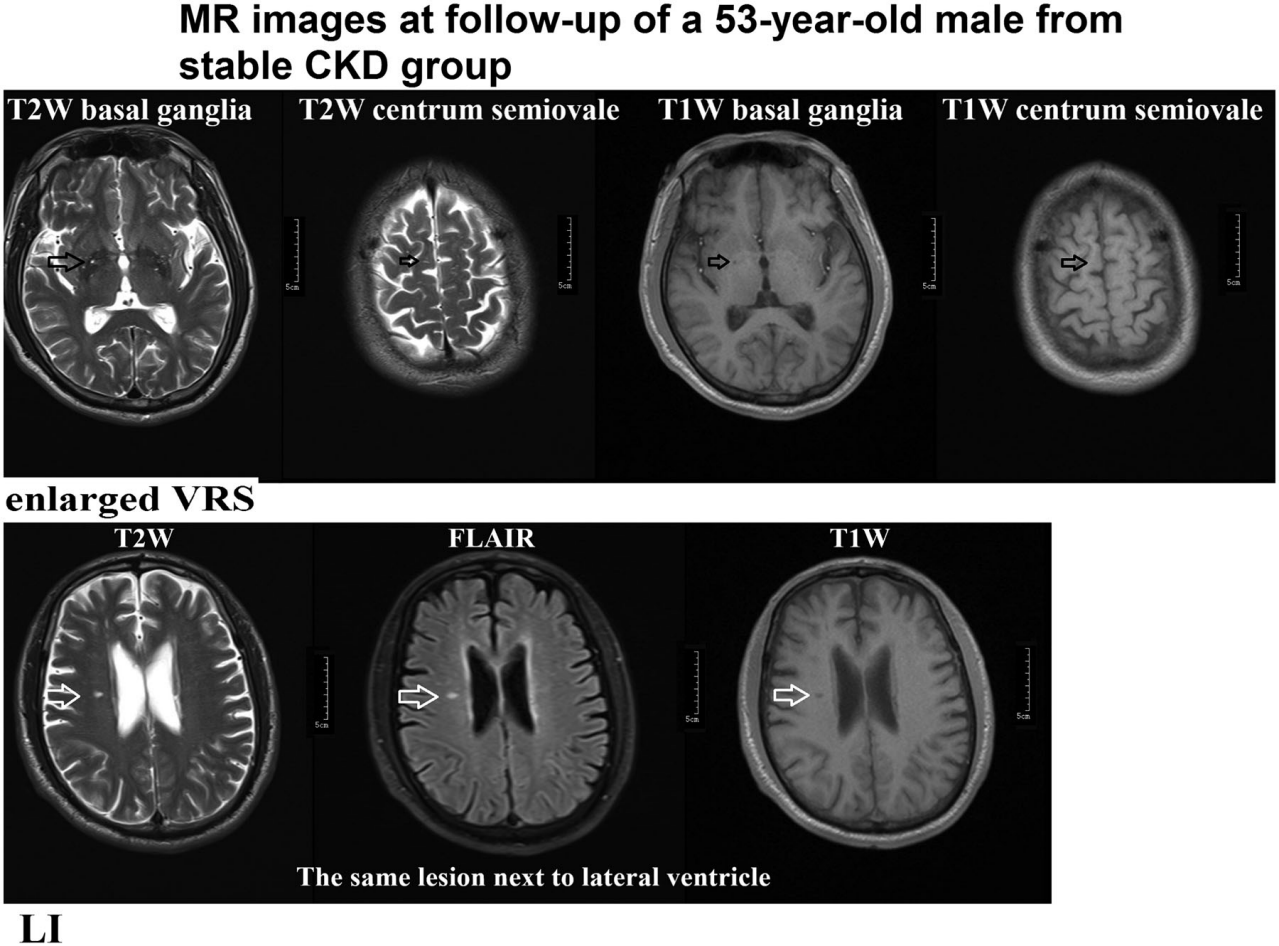
MR images from a 53-year-old male patient in the stable group at follow-up. Enlarged VRS (Virchow-Robin spaces) and LI (lacunar infarcts) lesions are shown. VRS hypersignal in T2W imaging and hyposignal in T1W images in the basal ganglia and centrum semiovale, VRS score 2 (according to the scale 0 = none, 1 = 1–10, 2 = 11–20, 3 = 21–40, and 4 = >40 by summing the basal ganglia and centrum semiovale VRS scores). LI was evaluated by hypersignal in T2/FLAIR images and hyposignal in T1W images located beside the lateral ventricle (white arrow).

In general, the diameter of a VRS is less than 2 mm. When the diameter of the surrounding space is greater than 3 mm, it is considered to be enlarged. This is consistent with the travel of the perforated vessels.VRSs are mainly found in three typical positions: type I is found along the artery in the basal ganglia, type II is along the medullary artery in the brain convexity and extending into the white matter below the cortex, and type III is found in the midbrain. Peripheral space around blood vessels on MRI show clear boundaries in sharp oval-, round-, or tubular-shaped structures (due to the location and type of cut—axial, sagittal, or coronal). The structures have the same signal as cerebrospinal fluid without enhancement or place effect (Figure 2).

We rated VRSs on T2 imaging (with all sequences available) in the basal ganglia and centrum semiovale as 0 = none, 1 = 1–10, 2 = 11–20, 3 = 21–40, and 4 = >40 enlarged perivascular spaces per side, using the higher side if they were asymmetric. We calculated total VRS scores (0–8) by summing the basal ganglia and centrum semiovale VRS scores (Figure 2) [2].

**Figure 3.**
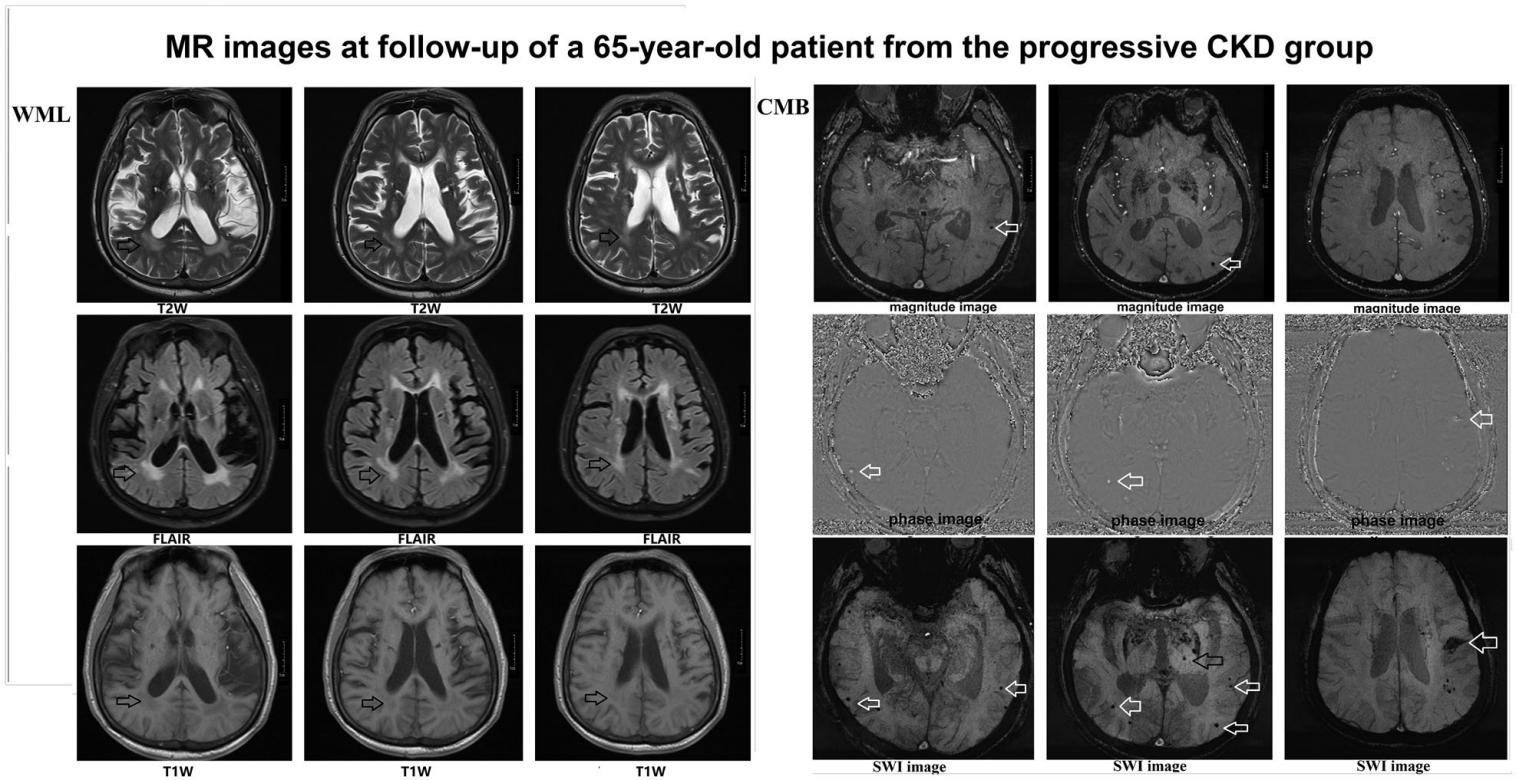
MR images from a 65-year-old patient from the progressive group at follow-up. WML (white matter lesions) and CMB (cerebral microbleeds) lesions are shown. WML surrounding the ventricle received Fazekas scale 2 points (fusion lesion) (T2W/FLAIR/T1W images) (black arrow). CMB lesions involved cortical, subcortical, and thalamic regions (white arrow) (magnitude image/phage image/SWI images).

A WML was defined as a signal abnormality of variable size in the white matter consisting of a hyperintensity on a T2-weighted image such as fluid-attenuated inversion recovery without cavitation, and decreased signal on T1-weighted images, according to previous studies (Figure 3) [2]. Fazekas scale (0–3 points) was used as follows: Grade 0 (normal), grade 1 (spot lesion), grade 2 (fusion lesion), and grade 3 (large fusion lesion) [4].

LI of presumed vascular origin was defined as a round or ovoid, subcortical, fluid-filled cavity, consistent with a small previously acute subcortical infarct or hemorrhage in the territory of a perforating arteriole (Figure 2).

A CMB on SWI MR images was defined as a 2–5 mm diameter uniform oval, low-signal focus with no surrounding edema. Investigators have proposed a specified rating scale for CMBs (Figure 3), the Microbleed Anatomical Rating Scale.

We classified microbleeds into ‘definite’ and ‘possible’ categories because a previous study suggested that such classification improves reliability. Definite microbleeds were defined as small, rounded or circular, well-defined hypointense lesions within brain parenchyma with clear margins ranging from 2 to 10 mm in size on SWI images; possible microbleeds were less well defined, less hypointense, or not strictly rounded or circular. The 2 to 10-mm size range includes the highest upper limit defined in previous studies [5].

Microbleeds were classified into deep, lobar, and infratentorial categories. Lobar MRI landmarks were defined according to Stark and Bradley and included cortical and subcortical regions (including subcortical U fibers). Deep regions included the basal ganglia, thalamus, internal capsule, external capsule, corpus callosum, and deep and periventricular white matter (DPWM); infratentorial regions included the brainstem and cerebellum. All regions were presented for easy reference in an anatomical diagram (drawn by S.M.G). The sum of definite and possible microbleeds was recorded as total microbleeds.

### Statistics

After passing the variance equality test, paired sample t-tests were used to compare the differences in VRS/WML/LI/CMB within the two groups, such as the difference between initial exam and follow-up for the progressive group, and the difference between initial exam and follow-up for the stable group (to the third decimal place). *p*-Values <0.05 were deemed statistically significant. Two-sample *t*-test of VRS/WML was used to compare the difference between progressive group and stable group at initial exam, and the difference between progressive group and stable group at follow-up. Chi-square tests were used to compare the differences between groups of LI/CMB. Pearson’s correlation was used to evaluate the relationship between eGFR and VRS, WML, LI, and CMB.

## Results

### Patient information

Of the 52 patients with CKD, 17 were classified into a progressive group and 35 were included in the stable group. There was no significant difference between the two groups in terms of age, gender, hypertension, diabetes and smoking. There were also no differences using Pearson correlation between VRS/WML/LI/CMB scores in terms of follow-up durations.

#### VRS

We did not observe a significant difference in VRS size between initial exam (group 1A) and follow-up (group 1B) for the progressive group (*p* = 0.083). Similarly, we did not observe a significant difference in VRS size between initial exam (group 2A) and follow-up (group 2B) for the stable group (*p* = 0.324)(Tables 2–5).

**Table 2.** MR evaluation of CSVD VRS (Virchow-Robin spaces) results and comparisons between two groups [progressive group at initial exam (group 1A), progressive group at follow-up (group 1B), stable group at initial exam (group 2A), stable group at follow-up (group 2B)].

VRS score	Mean ±SD
Group 1A (patients number)	1.647±0.996
Group 1B (patients number)	1.8240.8±83
Group 2A (patients number)	1.371±1.06
Group 2B (patients number)	1.429±1.037

Comparison between groups	*t* Value	*p* Value
Group 1B vs. Group 1A	−1.852	0.083
Group2B vs. Group2A	−1	0.324

**Table 3.** MR evaluation of CSVD WML (white matter lesions) results and comparisons between two groups [progressive group at initial exam (group 1A), progressive group at follow-up (group 1B), stable group at initial exam (group 2 A), stable group at follow-up (group 2B)].

WML score	Mean±SD
Group 1A (patients number)	0.647±0.702
Group 1B (patients number)	1.059±0.659
Group 2A (patients number)	0.657±0.765
Group 2B (patients number)	0.743±0.78

Comparison between groups	*t* Value	*p* Value
Group 1B vs. Group 1A	−3.347	0.004*
Group 2B vs. Group 2A	−1.785	0.083

*p < 0.05 was statistically significant.

**Table 4.** MR evaluation of CSVD LI (lacunar infarcts) results and comparisons between two groups [Progressive group at initial exam (group 1 A), Progressive group at follow-up (group 1B), Stable group at initial exam (group 2 A), Stable group at follow-up (group 2B)].

LI	Positive	Negative	Positive ratio (%)
Group 1A (patients number)	3	14	17.6
Group 1B (patients number)	4	13	23.5
Group 2A (patients number)	6	29	17.1
Group 2B (patients number)	7	28	20

Comparison between groups	*t* Value	*p* Value
Group 1B vs. Group 1A	−1	0.332
Group 2B vs. Group 2A	−1	0.324

**Table 5.** MR evaluation of CSVD CMB (cerebral microbleeds) results and comparisons between two groups [progressive group at initial exam (group 1A), progressive group at follow-up (group 1B), stable group at initial exam (group 2A), stable group at follow-up (group 2B)].

CMB	Positive	Negative	Positive ratio (%)
Group 1A (patients number)	0	17	0
Group 1B (patients number)	5	12	29.4
Group 2A (patients number)	0	35	0
Group 2B (patients number)	1	34	2.8

Comparison between groups	*t* Value	*p* Value
Group 1B vs. Group 1A	−2.582	0.020*
Group 2B vs. Group 2A	−1	0.324

*p < 0.05 was statistically significant.

#### WML

We observed a significant difference in WML between group 1A and group 1B (*p* = 0.004). No significant difference was observed between group 2A group 2B (*p* = 0.083) (Tables 2–5). WML in the progressive group were exacerbated at follow-up compared to at the initial exam (*p* = 0.004).

#### LI

No significant difference was observed between the progressive group at initial exam (group 1 A) and follow-up (group 1B), or between the stable group at initial exam (group 2A) and follow-up (group 2B) (*p* = 0.332 and 0.324, respectively) (Tables 2–5).

#### CMB

**Figure 4.**
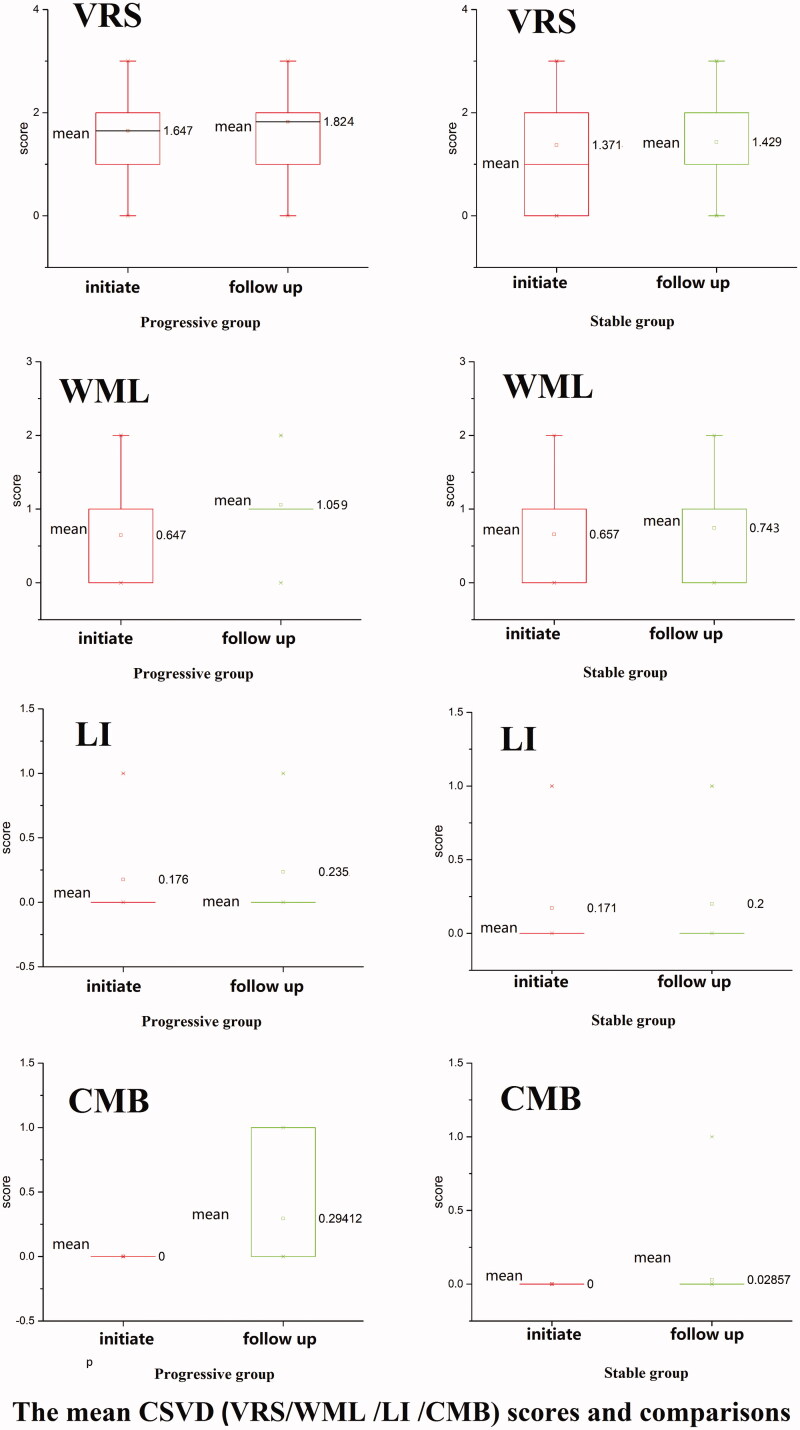
The mean CSVD scores of VRS (Virchow-Robin spaces)/WML (white matter lesions)/LI (lacunar infarcts)/CMB (cerebral microbleeds) and comparison between the two patient groups [progressive group at initial exam (group1A), progressive group at follow-up (group 1B), stable group at initial exam (group 2A), stable group at follow-up (group 2B)].

We observed a significant difference in presence of CMB between group 1A and group 1B (*p* = 0.020). No significant difference was observed between group 2A and group 2B (*p* = 0.324) (Figure 4, Tables 2–5).

There was no significant change in VRS, WML, LI, or CMB at initial exam compared to follow-up in the stable group. The presence of CMB were significantly different between the progressive group and stable group at follow-up (*p* = 0.004).

#### Pearson’s correlation for eGFR with VRS, WML, LI, and CMB

Only eGFR at follow-up was found to have a significant relationship with follow-up measures of VRS, WML, and CMB (correlation coefficients = −0.290, −0.284, −0.358, respectively; *p* = 0.037, 0.041, 0.009, respectively) (Tables 6 and 7).

**Table 6.** eGFR value and VRS/LI/WML/CMB comparisons between progressive group and stable group [progressive group at initial exam (group 1A), progressive group at follow-up (group 1B), stabilized group at initial exam (group 2A), stable group at follow-up (group 2B)].

	Number	eGFR	VRS	LI	WML	CMB	
		Mean + SD	Mean + SD	Mean + SD	Mean + SD	Mean + SD	
Group 1A	17	35.975 ± 11.645	1.647 ± 0.996	0.177 ± 0.393	0.647 ± 0.702	0	
Group 2A	35	36.765 ± 12.04	1.371 ± 1.06	0.171 ± 0.382	0.657 ± 0.765	0	
	*t* Value	−0.224	0.897	0.044	−0.046	–	
	*p* Value	0.823	0.374	0.965	0.964	–	
Group 1B	17	24.326 ± 9.217	1.824 ± 0.883	0.235 ± 0.437	1.059 ± 0.659	0.294	0.47
Group 2B	35	36.723 ± 11.416	1.429 ± 1.037	0.2 ± 0.406	0.722 ± 0.779	0.029	0.169
	*t* Value	−3.897	1.349	0.287	1.539	2.994	
	*p* Value	<0.001	0.183	0.775	0.13	0.004*	

*p < 0.05 was statistically significant.

**Table 7.** Pearson’s Correlation for eGFR with VRS, WML, LI, and CMB.

Correlation Coefficients	Initial eGFR	Follow-up eGFR	Initial VRS	Follow-up VRS	Initial WML	Follow-up WML	Initial LI	Follow-up LI	Initial CMB	Follow-up CMB
Initial eGFR	1	–	−0.234 (*p* = 0.095)	–	−0.196 (*p* = 0.164)	–	−0.144 (*p* = 0.307)	–	--	
Follow-up eGFR	–	1	–	−0.296* (*p* = 0.037)	–	−0.284 (*p* = 0.041)*	–	−0.181 (*p* = 0.2)		−0.358* (*p* = 0.009)

Two-tailed test of significance was used.

*Correlation is significant at the 0.05 level.

## Discussion

This study shows that with deterioration of renal function, as reflected in decreased eGFR, the patient group with progressive CKD had a greater degree of white matter degeneration and more CMB lesions. These observations suggest that there is a higher prevalence of cerebrovascular disease in the group with progressive CKD. CSVD in the progressive group was exacerbated as WML and CMB progressed.

A meta-analysis conducted by Lee et al. concluded that that the risk of stroke increased in subjects with an eGFR <60 mL/min/1.73 m^2^. Lower eGFR was a risk factor for both ischemic and hemorrhagic stroke. This study also found that when CKD patients with declining eGFR also had progressive cerebrovascular disease, CKD and CSVD may have been correlated; the greater the degree of intracranial lesion burden, the more eGFR decreased, suggesting that eGFR may be a predictor of ischemic or hemorrhagic stroke.

Features of CSVD include enlarged VRS and the presence of white matter lesions (WML), lacunar infarcts (LI), and cerebral microbleeds (CMB). It is widely accepted that chronic renal insufficiency is an independent risk factor for cerebrovascular disease [13,14]. In this study, the differences in CMB between progressive and stable CKD patient groups is clear. eGFR was significantly correlated with VRS, WML, and CMB at follow-up group (all participants, *n* = 52). This finding is consistent with a study by Xiao et al., who concluded that impaired estimated glomerular filtration rate is associated with severity of VRS enlargement [15].

### Relationship of VRS, LI, and renal function

In this study, VRS was significantly correlated with eGFR. LI was not significantly associated with eGFR. Previous studies have reported conflicting findings regarding the association between LI and eGFR, with some concluding that decreased eGFR tended to be associated with a higher prevalence of LI, while other studies did not support this interpretation.

### Relationship of WML and renal function

WML is one of manifestation of cerebrovascular disease, and is closely related to cerebral ischemia, stroke, degeneration, and many other neurologic diseases. Arntz et al. found that WMLs with intracranial merged lesion can reflect the severity of CSVD in the elderly, and the development of WMLs can be regarded as the terminal changes of CSVD prognosis. Chronic kidney disease has been reported to be associated with LI, WML, and CMB. The results of the present study are consistent with these conclusions, specifically that as renal function declines, the greater the degree of white matter degeneration, and the higher the incidence of CSVD.

Possible explanations underlying the observed relationship between CKD and CSVD include: (1) microvasculature of the kidney and brain are similar in structure and function, as well as in microvascular regulation; (2) the vascular resistance of both organs is low, thus allowing high blood volumes to perfuse continuously; (3) both organs are particularly susceptible to atherosclerosis; (4) kidney damage is characterized by abnormalities in glomerular endothelial cell function, and WML is caused by endothelial cell dysfunction; (5) when chronic kidney disease progresses, associated factors such as accumulation of toxins (e.g. urate), anemia, malnutrition, sodium and water retention, and abnormal calcium and phosphorus regulation may contribute to cerebrovascular disease. Related to endothelial dysfunction, one study found that Klotho, a protein expressed by the distal vessels of the kidney, is decreased in chronic kidney disease, leading to abnormal vascular endothelial function and cerebrovascular disease.

### Relationship of CMB and renal function

Pathologic-radiologic correlation studies have revealed that CMBs are focal accumulations of hemosiderin adjacent to abnormal blood vessels demonstrating fibrolipohyalinosis or amyloid microangiopathy. CMBs are the radiologic correlates of extravasation of blood components through injured or fragile vascular walls, or of frank small hemorrhage spots [16,17]. In addition, the present study found that there is a significant difference in CMB between patients with progressive CKD (progressive group) and patients with stable CKD (stable group). We used SWI sequence to detect the occurrence and progression of CMB; its sensitivity and accuracy has been widely recognized [18,19].

Brain atrophy is sometimes considered another potential MRI feature of CSVD. Although studies have suggested an association between brain atrophy and CSVD, brain atrophy is not specific to CSVD, often occurring in many other conditions. Therefore, our study did not include brain atrophy as a consideration for the detection or assessment of CSVD.

The results of this study support a relationship between the deterioration of renal function and cerebrovascular disease, but the mechanism underlying this relationship is not clear and needs further research. The follow-up time in this study may have been too short to fully evaluate and detect relevant changes in LI and VRS. Further study is required to clarify these issues.

In conclusion, imaging findings suggest that there is a significant relationship between patients with progressive CKD and CSVD, especially as indicated by VRS, WML, and CMB, although the mechanism underlying this relationship remains to be understood. VRS, WML, and CMB significantly correlated with eGFR, suggesting that eGFR may not only contribute to the evaluation of renal function in patients with CKD but may also find utility in the prediction of cerebral small vessel disease. eGFR may reflect a more generalized process indicative of underlying vascular damage, not limited solely to the kidney.

It is a bold speculation that when CSVD progresses, renal function in patients with CKD may correspondingly decline. Conversely, when renal function in patients with CKD declines, CSVD may also progress and require more careful attention. These speculations remain for future studies.
